# Supplementary material for: Identification of photoperiod-induced specific miRNAs in the adrenal glands of Sunite sheep (Ovis aries)
Source: Front Vet Sci. 2022 Jul 22;9:888207. doi: 10.3389/fvets.2022.888207 (PMC9354845; doi:10.3389/fvets.2022.888207)
Supplement: Supplementary file 1 [file Data_Sheet_1.ZIP › Supplementary file/miRDeep2 file.zip!/miRDeep2 file/Know_Structure/LP42A3/oar-mir-30b.pdf]

miRBase precursor : oar-mir-30b
Total read count : 27769
oar-miR-30b read count : 27521
remaining reads : 248

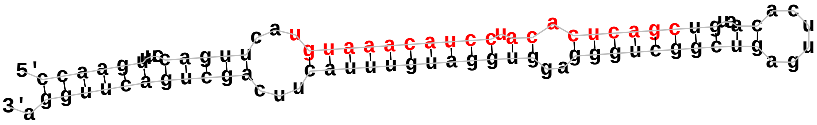

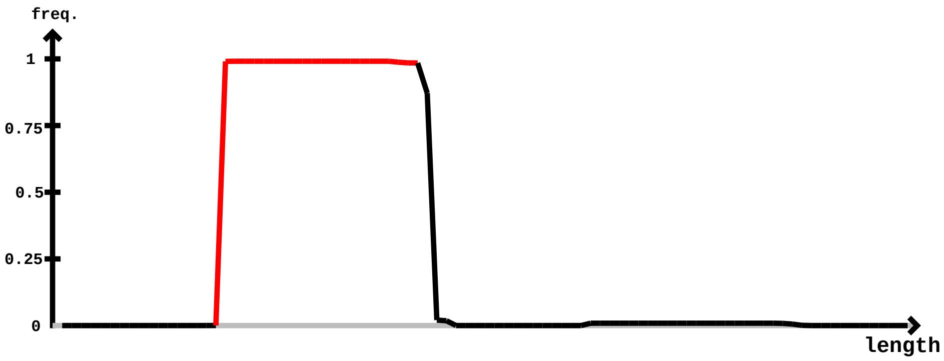

oar-miR-30b

```
5'- ccaaguuuccaguucauguaaacauccuacacucagcuguaacacuugagucggcugggagguggauguuuacuucagcugacuugga   -3'    exp
    ((((((...(((((...((((((((((.((.((((((((..((......))))))))))..))))))))))))...))))))))))).    reads      mm         sample
    ..............cauguaaacauccuacacucagcu..................................................    3          0          seq
    ...............auguaaacauccuacacucagc...................................................    1          0          seq
    ...............auguaaacauccuacacucagcu..................................................    4          0          seq
    ................uguaaacauccuacacuU......................................................    20         1          seq
    ................uguaaacauccuacacuc......................................................    78         0          seq
    ................uguaaacaCccuacacuc......................................................    2          1          seq
    ................uguaaacauccuacacuca.....................................................    69         0          seq
    ................uguaaacauccuacacucag....................................................    11         0          seq
    ................uguaaacauccuacacucagc...................................................    3136       0          seq
    ................uguaaacauccuacacucagcu..................................................    23615      0          seq
    ................uguaaacauccuacacucagcug.................................................    69         0          seq
    ................uguaaacauccuacacucagcugu................................................    498        0          seq
    ................uguaaacauccuacacucagcugua...............................................    2          0          seq
    .................guaaacauccuacacucagcu..................................................    13         0          seq
    ......................................................cugggagguggauguuuacu..............    20         0          seq
    ......................................................cugggagguggauguuuacuu.............    71         0          seq
    ......................................................cugggagguggauguuuacuuc............    130        0          seq
    ......................................................cugggagguggauguuuacuuca...........    23         0          seq
    .......................................................ugggagguggauguuuacu..............    1          0          seq
    .......................................................ugggagguggauguuuacuu.............    1          0          seq
    .......................................................ugggagguggauguuuacuuc............    1          0          seq
    .......................................................ugggagguggauguuuacuuca...........    1          0          seq
```
